# Data collection tools for maternal and child health in humanitarian emergencies: a systematic review

**DOI:** 10.2471/BLT.14.148429

**Published:** 2015-06-24

**Authors:** Thidar Pyone, Fiona Dickinson, Robbie Kerr, Cynthia Boschi-Pinto, Matthews Mathai, Nynke van den Broek

**Affiliations:** a Centre for Maternal and Newborn Health, Liverpool School of Tropical Medicine, Pembroke Place, Liverpool, E3 5QA, England.; b Department of Maternal, Newborn, Child & Adolescent Health, World Health Organization, Geneva, Switzerland.

## Abstract

**Objective:**

To describe tools used for the assessment of maternal and child health issues in humanitarian emergency settings.

**Methods:**

We systematically searched MEDLINE, Web of Knowledge and POPLINE databases for studies published between January 2000 and June 2014. We also searched the websites of organizations active in humanitarian emergencies. We included studies reporting the development or use of data collection tools concerning the health of women and children in humanitarian emergencies. We used narrative synthesis to summarize the studies.

**Findings:**

We identified 100 studies: 80 reported on conflict situations and 20 followed natural disasters. Most studies (76/100) focused on the health status of the affected population while 24 focused on the availability and coverage of health services. Of 17 different data collection tools identified, 14 focused on sexual and reproductive health, nine concerned maternal, newborn and child health and four were used to collect information on sexual or gender-based violence. Sixty-nine studies were done for monitoring and evaluation purposes, 18 for advocacy, seven for operational research and six for needs assessment.

**Conclusion:**

Practical and effective means of data collection are needed to inform life-saving actions in humanitarian emergencies. There are a wide variety of tools available, not all of which have been used in the field. A simplified, standardized tool should be developed for assessment of health issues in the early stages of humanitarian emergencies. A cluster approach is recommended, in partnership with operational researchers and humanitarian agencies, coordinated by the World Health Organization.

## Résumé

**Objectif:**

**Décrire les outils utilisés pour évaluer les problèmes en matière de santé maternelle et infantile dans les situations d'urgence humanitaire.**

**Méthodes:**

**Nous avons recherché de façon systémique, dans les bases de données Medline, Web of Knowledge et Popline, les études publiées entre janvier 2000 et juin 2014. Nous avons également fait des recherches sur les sites Internet d'organisations intervenant dans les situations d’urgence humanitaire. Nous avons inclus les études qui se rapportaient au développement ou à l'utilisation d'outils de collecte de données concernant la santé des femmes et des enfants dans des situations d'urgence humanitaire. Nous avons résumé ces études par une synthèse narrative.**

**Résultats:**

**Nous avons retenu 100 études: 80 portaient sur des situations de conflit et 20 faisaient suite à des catastrophes naturelles. La plupart de ces études (76/100) s'intéressaient à la situation sanitaire des populations affectées tandis que 24 d'entre elles s'intéressaient à la disponibilité de services de santé et à leur couverture. Sur 17 outils de collecte de données identifiés, 14 concernaient la santé sexuelle et génésique, neuf la santé de la mère, du nouveau-né et de l'enfant, et quatre servaient à recueillir des informations sur la violence sexuelle ou exercée à l'égard des femmes. Soixante-neuf études avaient été réalisées à des fins de suivi et d'évaluation, dix-huit de sensibilisation, sept pour la recherche opérationnelle et six pour évaluer les besoins.**

**Conclusion:**

**Des moyens pratiques et efficaces de collecte de données sont nécessaires pour orienter les actions permettant de préserver des vies humaines dans les situations d'urgence humanitaire. Il existe une grande variété d'outils disponibles, dont tous n'ont pas été employés sur le terrain. Il faudrait développer un outil simplifié et standardisé pour évaluer les problèmes sanitaires dès les premières phases des urgences humanitaires. Il est recommandé d'adopter une approche groupée, en partenariat avec les chercheurs opérationnels et les agences humanitaires, sous la coordination de l'Organisation mondiale de la Santé.**

## Resumen

**Objetivo:**

Describir las herramientas utilizadas para evaluar los problemas de salud materna e infantil en entornos de emergencias humanitarias.

**Método:**

Se realizaron búsquedas sistemáticas en las bases de datos de Medline, Web of Knowledge y Popline para encontrar estudios publicados entre enero de 2000 y junio de 2014. También se realizaron búsquedas en páginas web de organizaciones activas en emergencias humanitarias. Se incluyeron estudios que informaban sobre el desarrollo o el uso de herramientas de recopilación de datos relacionadas con la salud de las mujeres y los niños durante emergencias humanitarias. Se utilizó la síntesis narrativa para resumir los estudios.

**Resultados:**

Se identificaron 100 estudios: 80 informaban sobre situaciones de conflicto y 20 sobre desastres naturales. La mayoría de los estudios (76/100) se centraban en el estado de la salud de la población afectada, mientras que 24 lo hacían en la disponibilidad y cobertura de los servicios de salud. De las 17 herramientas de recopilación de datos diferentes identificadas, 14 se centraban en la salud reproductiva y sexual, nueve trataban sobre salud maternal, neonatal e infantil y cuatro se utilizaban para recopilar información sobre violencia sexual o basada en el género. 69 estudios se habían realizado con fines de supervisión y evaluación, 18 para promoción, siete para investigaciones operacionales y seis para la evaluación de necesidades.

**Conclusión:**

Se necesitan medios prácticos y efectivos de recopilación de datos para informar de acciones para salvar vidas en emergencias humanitarias. Existe una amplia variedad de herramientas disponibles, y no todas se han utilizado en este campo. Se debería desarrollar una herramienta simplificada estándar para evaluar los problemas de salud en las primeras etapas de emergencias humanitarias. Se recomienda un enfoque por grupos en cooperación con investigadores operacionales y agencias humanitarias, coordinados por la Organización Mundial de la Salud.

## ملخص

**الغرض:**

وصف الأدوات المستخدمة في تقييم مشكلات صحة الأمومة والطفولة في أماكن وقوع الحالات الإنسانية الطارئة.

**الطريقة:**

قمنا بإجراء بحث منهجي في قواعد معطيات Medline، وWeb of Knowledge، وPopline للدراسات المنشورة في الفترة من يناير/كانون الثاني 2000 وحتى يونيو/حزيران 2014، كما بحثنا أيضًا في المواقع الإلكترونية للمؤسسات الفاعلة في مجال الحالات الإنسانية الطارئة. وقمنا بتضمين دراسات توضح إعداد أدوات جمع البيانات أو استخدامها فيما يتعلق بصحة النساء والأطفال في الحالات الإنسانية الطارئة. كما اتبعنا أسلوبًا تجميعيًا سرديًا لتلخيص الدراسات.

**النتائج:**

لقد حددنا 100 دراسة، فوجدنا أن 80 دراسة منها أوردت بيانات عن حالات الصراع فيما تابعت 20 دراسة منها وقوع كوارث طبيعية. ركزت معظم الدراسات (76 من إجمالي 100) على الحالة الصحية للشريحة السكانية المتأثرة، في حين ركزت 24 دراسة على مدى توافر الخدمات الصحية ونطاق تغطيتها. ومن ضمن 17 أداة من أدوات جمع البيانات المختلفة التي تم تحديدها، ركزت 41 أداة على الصحة الجنسية والإنجابية، وكانت تسع أدوات معنية بصحة الأم والوليد والطفل، فيما تم استخدام أربع أدوات لجمع المعلومات عن العنف الجنسي أو العنف الجنساني. تم إجراء تسعة وستين دراسة لأغراض الرصد والتقييم، فيما تمت 18 دراسة للمناصرة، وسبع دراسات للبحوث الميدانية، وست دراسات لتقييم الاحتياجات.

**الاستنتاج:**

هناك حاجة لاتباع وسائل عملية وفعالة لجمع البيانات اللازمة التي تقوم على أساسها الإجراءات الحاسمة لإنقاذ الأرواح في الحالات الإنسانية الطارئة. وتتوفر مجموعة متنوعة وواسعة من الأدوات، والتي لم يتم استخدامها جميعًا في الميدان. وينبغي إعداد أدوات مبسطة وموحدة لتقييم المشكلات الصحية في المراحل الأولى من الحالات الإنسانية الطارئة. يُوصى باتباع نهج قطاعي (Cluster approach)، بالاشتراك مع الباحثين التنفيذيين ووكالات المساعدة الإنسانية، وبالتنسيق من جانب منظمة الصحة العالمية.

## 摘要

**目的:**

旨在描述在人道主义紧急情况中用于评估孕产妇和儿童健康问题的工具。

**方法:**

我们在联机医学文献分析和检索系统 (Medline)、Web of Knowledge 和  Popline 数据库中系统搜索了于  2000 年  1 月至  2014 年  6 月之间发表的研究报告。我们还搜索了在人道主义紧急情况中表现积极的组织的网站。 我们涵盖的研究报告了对与人道主义紧急情况中的妇女和儿童有关的数据收集工具的开发和使用。 我们采用叙述性综合法对研究进行了总结概括。

**结果:**

我们确定了 100 项研究： 其中 80 项报告了冲突局势，其余 20 项报告了自然灾害。 大部分研究 (76/100) 侧重于受灾人群的健康状态，而其他 24 项研究侧重于卫生服务的可用性和覆盖范围。 在确定的 17 种不同的数据收集工具中，14 种侧重于性与生殖健康，九种与孕产妇、新生儿和儿童健康有关，四种用于收集与性或性暴力行为有关的信息。 69 项研究是以监控和评估为目的而开展的，18 项以宣传倡导为目的，7 项以操作性研究为目的，6 项以需求评估为目的。

**结论:**

我们需要采取实用、有效的数据收集方式，以了解人道主义紧急情况中的生命拯救行动。 可用工具各式各样，然而并非所有工具都曾用于该领域。 我们应开发出标准化的简易工具，用于评估在人道主义紧急情况早期出现的健康问题。 建议采用聚类的方法，并且在世界卫生组织的协调下与操作性研究人员及人道主义机构开展合作。

## Резюме

**Цель:**

Описать инструменты, используемые для оценки проблем материнского здоровья и здоровья детей в условиях чрезвычайной ситуации гуманитарного характера.

**Методы:**

Был проведен систематический поиск исследований в базах данных Medline, Web of Knowledge и Popline, опубликованных с января 2000 года по июнь 2014 года. Поиск также осуществлялся на веб-сайтах организаций, работающих в условиях чрезвычайных ситуаций гуманитарного характера. В обзор были включены исследования, в которых сообщалось о разработке или использовании инструментов сбора данных о здоровье женщин и детей в условиях чрезвычайных ситуаций гуманитарного характера. Для получения сводных данных по этим исследованиям использовался нарративный синтез.

**Результаты:**

Нами было выявлено 100 исследований: в 80 из них сообщалось о конфликтах, а в 20 речь шла о стихийных бедствиях. Большая часть исследований (76 из 100) была посвящена состоянию здоровья затронутого бедствием населения, а в 24 речь шла о доступности услуг здравоохранения об охвате населения такими услугами. Из 17 выявленных инструментов сбора данных 14 касались сексуального и репродуктивного здоровья, девять — здоровья матерей, новорожденных и детей, четыре опроса использовались для сбора информации о сексуальном насилии или насилии по половому признаку. Шестьдесят девять исследований были проведены с целью мониторинга и оценки ситуации, 18 — из соображений защиты прав человека, семь — в порядке операционных исследований, шесть — для оценки потребностей.

**Вывод:**

Практичные и эффективные инструменты сбора данных оказываются необходимыми для мероприятий по спасению жизни, предпринимаемых в ходе чрезвычайных ситуаций гуманитарного характера. Доступно множество инструментов, однако не все они используются на практике. Следует разработать упрощенное стандартизированное средство оценки проблем со здоровьем на ранних этапах чрезвычайных ситуаций гуманитарного характера. Рекомендуется использовать кластерный подход и взаимодействовать с гуманитарными организациями и специалистами по операционным исследованиям при координации со стороны Всемирной организации здравоохранения.

## Introduction

Humanitarian emergencies are natural disasters, man-made events or a combination of both that represent critical threats to the health, safety, security or wellbeing of a community.^1^ Humanitarian emergencies resulting from conflict, natural disasters, famine or communicable disease outbreaks have important health implications. Currently, there are approximately 39 million people displaced by conflict or violence.^2^ Every year, millions are displaced due to weather-related or geophysical disasters.^3^ Women and children are generally the worst affected – representing over three-quarters of the estimated 80 million people in need of humanitarian assistance in 2014.^4^^,^^5^ Moreover, many countries with high maternal, newborn and child mortality rates are affected by humanitarian emergencies.

Humanitarian emergencies are frequently characterized by the collapse of basic health services. For better decision-making, coordination and response in such emergencies, humanitarian actors need access to appropriate information.^4^^,^^6^^,^^7^ Studies have reported that during humanitarian emergencies, there can be either a shortage or, conversely, an overload of information. Both situations impair provision of effective humanitarian assistance.^8^

Sexual and reproductive health has historically been neglected in humanitarian emergency settings.^9^ Health services provided for women and children vary depending on location, climate, culture, existing infrastructure, population health and type of humanitarian crisis. The types of response also vary, with multiple governments and humanitarian agencies involved. Efficient, easy to use, comprehensive data collection tools are needed to aid situation analysis, decision-making and coordination of responses to humanitarian crises.^10^

We review tools for collection of data concerning the health of women and children in humanitarian emergencies. We identify which tools are available and where they have been used. For each study, we describe the setting and purpose of the study, the types of data collected and the tools used to collect the data.

## Methods

### Search strategy

We conducted a systematic review according to current guidelines.^11^ We searched MEDLINE, Web of Knowledge and POPLINE databases for studies in English published between 1 January 2000 and 30 June 2014. Searches incorporated medical subject heading terms, keywords and free text using the following search terms: “reproductive health”, “sexual”, “maternal”, “newborn”, “child/child health service*”, “pregnan*”, “neonat*” under one search string and “disaster”, “post conflict”, “war”, “humanitarian”, “refugee”, “internally displaced” under another string. The Boolean operator “OR” was used for the terms under each search string and “AND” was used to combine the two strings. The detailed search strategy is available from the authors.

Through a snowballing process, we identified organizations known for their work in humanitarian emergencies and searched the websites of these organizations – including CARE International, the Centers for Disease Control and Prevention, Harvard Humanitarian Initiative, the Inter-Agency Standing Committee, the International Federation of Red Cross and Red Crescent Societies (IFRC), the Joint United Nations Programme on HIV/AIDS (human immunodeficiency virus/acquired immunodeficiency syndrome), Knowledge for Health (K4Health), Médecins Sans Frontières (MSF), the Office of the United Nations High Commissioner for Refugees, Oxfam, the Reproductive Health Response in Crises Consortium, Save the Children, the United Nations Population Fund (UNFPA), the Women’s Refugee Commission, the World Health Organization (WHO) and World Vision. The snowballing process was carried out using the reference list of included studies and the organizations known for humanitarian emergencies. We also searched the references and authors of all included studies.

### Inclusion and exclusion criteria

**Fig. 1:**
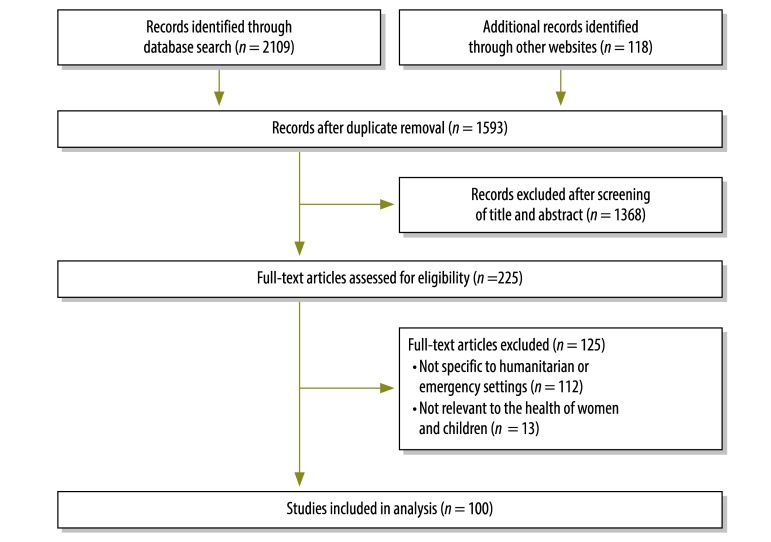
Flowchart for the selection of studies: data collection tools for maternal and child health in humanitarian emergencies

Studies were included if they reported the development or use of data collection tools concerning the health of women and children in a humanitarian emergency. We included studies, even when tools for data collection were not specified or the method was not described (Fig. 1).

Two authors independently searched databases and websites. The titles and abstracts of identified studies were screened and excluded if not meeting the inclusion criteria. Full texts of remaining studies were assessed for eligibility. When it was not clear if a study should be included or not, two reviewers discussed the study and if consensus was not reached, a third reviewer was consulted. The reviewers summarized information on tools used, type of data collected and the purpose of the study. Data were classified into four categories, based upon the continuum of care: (i) sexual and reproductive health including sexual/gender-based violence and family planning; (ii) maternal and neonatal health; (iii) infant and child health; and (iv) sexually transmitted infections, including HIV/AIDS.

Studies that met the inclusion criteria were summarized using textual narrative synthesis.^10^ First, we developed a commentary report on the type and characteristics of the included studies, context and findings using a standard matrix. The reviewers then looked for similarities and differences among studies to discuss and draw conclusion across the studies.

## Results

We identified 2227 studies: 2109 publications from databases and 118 studies from websites. After removal of duplicates, the titles and abstract of 1593 studies were screened and of these, 225 studies were identified as eligible for full text review. Of these, 112 were not specific to humanitarian or emergency settings and 13 were not relevant (Fig. 1).

Of the 100 studies identified, 69 studies described the number of people affected. The population consisted of 677 568 individuals; 65 971 were identified as women and 57 427 children; 37 660 (57%) of children were younger than five years (Table 1, available at: http://www.who.int/bulletin/volumes/93/9/14-148429). Studies ranged in sample size from seven (in case studies of survivors of sexual violence)^63^ to 179 172 (in a rapid assessment of micronutrient deficiency following drought).^71^ Eighty studies reported on conflict situations, while 20 studies reported on situations following a natural disaster (tsunami, hurricane or drought). Nineteen studies reported on the timing of data collection: three studies collected data within one week,^70^^,^^72^^,^^79^ five within three months,^7^^,^^19^^,^^49^^,^^51^^,^^52^ and 11 studies collected data six months to one year after the onset of the humanitarian emergency.^21^^,^^36^^,^^38^^,^^46^^,^^55^^,^^60^^,^^73^^,^^76^^,^^81^^,^^86^^,^^87^

**Table 1:** Summary table of included studies by author

Author	Tools and methods	Type of data collected by category	Outcome (use of data collected)	Setting (country – type of emergency if information available)	Populations included	Publication type
Abdalla et al., 2008^12^	Cross-sectional survey; interviews and physical assessments	Maternal and neonatal health; Infant and child health Anthropometric measures including haemoglobin level, diarrhoea and ARI and the feeding practices of mothers	Prevalence of malnutrition, cumulative incidence of diarrhoea and ARI and the feeding practices of mothers	Nepal – refugees from Bhutan	413 women of reproductive age and 497 children younger than five years	Not peer reviewed
Abdeen et al., 2007^13^	Validated multistage clustered design using an interviewer-administered questionnaire and anthropometric measurements	Infant and child health Basic demography, feeding patterns, food availability, dietary intake and anthropometric measurements	Assessment of nutritional status of children aged 6 month to 5 years following food assistance	West Bank and Gaza strip – uprising	3089 children younger than five years	Peer reviewed
Abu Mourad et al., 2004^14^	Cross-sectional household survey	Infant and child health Data on socioeconomic, environmental health, hygiene, incidence of intestinal parasites and diarrhoea by age segregation	Causes of gastrointestinal illness in refugee camp	West Bank and Gaza strip	1625 women of reproductive age	Peer reviewed
Amowitz et al., 2002^15^	Cross-sectional randomized survey	SRH including GBV Physical and mental health perception, personal experiences on sexual assault and human rights abuse	Estimate of war and non-war sexual violence against Internally Displace Person and non-Internally Displaced women	Sierra Leone – IDP	991 women	Peer reviewed
Annan et al., 2008^16^	Household surveys	SRH including GBV Long-term effects of abduction, war violence, forced marriage and motherhood on young women and girls	Basis for advocacy to recognize the importance of the problem	Uganda - protracted internal war	619 young women and girls	Not peer reviewed
ARC International, 2003^17^	Baseline survey results compared with post-intervention survey	STI including HIV Knowledge, attitudes and behaviour regarding HIV/AIDS and other STIs before and after intervention	To formulate policy recommendations	Sierra Leone	956 individuals	Not peer reviewed
Armony-Sivan et al., 2013^18^	Cross-sectional survey, interview-based study using regression analysis	Maternal and neonatal health Maternal data on basic sociodemographics including ANC and PNC Maternal depression and anxiety	To examine the relationship between maternal stress in early pregnancy and cord-blood ferritin concentration	Southern Israel – post-emergency (after rocket attack during the military operation)	140 pregnant women	Peer reviewed
Arques et al., 2013^19^	Cross-sectional, secondary data from a hospital	Infant and child health Demographic, physical, microbiologic findings, treatment and outcomes of children	To analyse the results of clinical and microbiological characteristics of children treated in the hospital	Haiti – earthquake 2010	118 individuals, 53 children	Peer reviewed
Assefa et al., 2001^20^	Two-stage cluster household survey, standardized data collection tool	Infant and child health Weight for age data of children younger than five years, food coping mechanisms	Causes of crude and under 5 mortality rates and prevalence of malnutrition	Afghanistan – civil war and drought	3165 individuals of which 41% (763) children younger than five years	Peer reviewed
Ayoya et al., 2013^21^	Daily data recording of attendees managed using standardized form	Maternal and neonatal health; Infant and child health Feeding practices and anthropometric measurements	To evaluate methods and guidelines on implementation of baby tents to facilitate breast feeding following natural disasters	Haiti – earthquake	180 499 mother-infant pairs, 52 503 pregnant women	Peer reviewed
Baines, 2014^22^	Cross-sectional, qualitative data using FGD	SRH including GBV Perceptions of former commanders and wives on historical evolution of forced marriage	To highlight strategic use of sexual violence in political projects	Sudan – post-conflict	18 participants of which 15 are women	Peer reviewed
Balsara et al., 2010^23^	Interviewer-administered questionnaire, physical examination and lab tests	SRH including GBV Knowledge on RTIs and behavioural factors contributing to RTIs	Prevalence of RTI in Afghan refugee women	Pakistan – refugee camps	634 women of reproductive age	Peer reviewed
Bartels et al., 2010^24^	Retrospective review of medical records using non-systematic convenience sample; semi-structured interviews with an open self-reporting interview	SRH including GBV Physical and psychological consequences of sexual violence	To describe the demographics and define both physical and psychosocial consequences of sexual violence	Democratic Republic of the Congo – ongoing prolonged conflict	1021 women of which 82.7% are women of reproductive age	Peer reviewed
Bartels et al., 2013^25^	Retrospective analysis of secondary data	SRH including GBV Perpetrator profiles; attack characteristics including type and location of sexual violence	To describe the patterns of sexual violence described by the survived victims and analyse perpetrator profiles	Democratic Republic of the Congo – post conflict	NA	Peer reviewed
Bbaale, 2011^26^	Two–stage cluster using Uganda Demographic and Health Survey (2006)	Infant and child health Prevalence of diarrhoea and ARI	Factors associated with occurrence of diarrhoea and incidence ofARI in children younger than five years	Uganda - IDP camps	NA	Peer reviewed
Bbaale & Guloba, 2011^27^	Two-stage cluster using Uganda Demographic and Health Survey (2006)	Maternal and neonatal health; Infant and child health Factors (maternal education, community infrastructure, occupation, location, wealth, religion and age) associated with utilization of professional childbirth care	To improve uptake of skilled care at birth	Uganda - IDP camps	NA	Peer reviewed
Beatty et al., 2001^28^	Interviews with IDP and health staff; no specific tool described	SRH including GBV; Maternal and neonatal health; STI including HIV RH needs and services available	To assess the RH needs and RH services available	Angola – IDP in civil war	NA	Not peer reviewed
Bilukha et al., 2007^29^	Victim data collection, demographics and standard international management system for mine action data collection form	Infant and child health Children are included as demographic indicators under landmine injuries	Rates of injury from landmines in civilians	Chechnya, Russia – armed conflict	NA	Peer reviewed
Bisimwa et al., 2009^30^	Community based child nutritional monitoring, physical assessment	Infant and child health Weight for age measurement, incidence of childhood illnesses	Assessment of effectiveness of monitoring the growth of pre-school children from a cohort of endemic malnutrition	Democratic Republic of the Congo – armed conflict	5479 children younger than five years	Peer reviewed
Brown et al., 2010^31^	Population based study, laboratory tests and demographic data	Infant and child health Data on blood lead level and chelation	Association between lead poisoning prevention activities and blood lead levels among children	Serbia – IDP camp	145 children	Peer reviewed
Burns et al., 2012^32^	Clinical questionnaire based on the integrated management of childhood illness	Infant and child health Prevalence of malaria among children	Development of a novel tool to control malaria in an emergency setting	Sierra Leone – refugee camp	222 children aged 4–36 months	Peer reviewed
Callands et al., 2013^33^	Secondary data analysis of DHS data	SRH including GBV IPV experiences, attitude towards IPV, ability to negotiate safe sex and STIs incidence	To identify the relationship between STIs and negotiation for sexual safety with intimate partners among young women	Liberia – post-conflict	NA	Peer reviewed
Casey et al., 2009^34^	Facility assessments, interviews, observation and clinical record review	Maternal and neonatal health Assessment of RH facilities	To determine availability, utilization and quality of emergency obstetric care and family planning services to avert death and disability	Democratic Republic of the Congo – conflict	NA	Peer reviewed
Casey et al., 2013^35^	Population based baseline and end-line surveys; CDC’s Reproductive health assessment toolkit for conflict	SRH including GBV Family planning	To evaluate the effectiveness of provision of long acting family planning methods both in mobile clinic and health centres	Northern Uganda	1778 women of reproductive age	Peer reviewed
CDC, 2001^36^	Three-stage cluster sample design; interview and physical assessments	Infant and child health Anthropometric measures including haemoglobin level	Determination of causes of malnutrition (acute and chronic)	Mongolia – severe winter weather	937 children aged between 6–59 months	Not peer reviewed
D’Errico et al., 2013^37^	Semi-structured interviews from 16 locations from male and female respondents	SRH including GBV; Maternal and neonatal health Local perceptions of the determinants of maternal health; Women’s coping mechanisms regarding barriers to healthcare; existence of informal systems of social support	Some understanding of social determinants of health	Four eastern provinces of Democratic Republic of the Congo	121 respondents	Peer reviewed
Doocy et al., 2009^38^	Two-stage cluster design, survey instrument not specified	Maternal and neonatal health; Infant and child health	Information on pre- and post-tsunami household composition, including deaths and injuries	Indonesia – tsunami	NA	Peer reviewed
Dossa et al., 2013^39^	Cross-sectional population-based study	SRH including GBV; STI including HIV Fistula , chronic pelvic pain, desire for sex and desire for children	To investigate the relationship between sexual violence and serious RTIs including fistula	Democratic Republic of the Congo – post-conflict	7935 individuals	Peer reviewed
Dua et al., 2013^40^	Retrospective analysis using data from military hospitals in Baghdad	Infant and child health Demographic and physiologic data on paediatric vascular injuries	To describe the experience of paediatric vascular injuries in a military combat support hospital	Iraq – post conflict	320 females	Peer reviewed
Edwards et al., 2013^41^	Cross-sectional analysis of hospitals admission databases	Infant and child health % of children required transfusion, location of injury, length of hospital stay and in-hospital mortality	To define the scope of combat and noncombat-related inpatient paediatric humanitarian care provided by the military of the USA	Afghanistan and Iraq – post-conflicts	NA	Peer reviewed
Elhag et al., 2013^42^	Cross-sectional analysis using clinical data	Infant and child health Clinical history, sociodemographic characteristics, physical examination and laboratory tests of diarrhoea among children	To determine prevalence of rotavirus and adenovirus associated diarrhoea	Sudan – IDP	NA	Peer reviewed
Falb et al., 2014^43^	Cross-sectional interview-based survey	SRH including GBV; Maternal and neonatal health Frequencies of pregnancy complications, violence, conflict victimization	To guide maternal health programmatic efforts among refugee women	Border between Myanmar and Thailand – refugee camps	710 individuals (330 children younger than five years)	Peer reviewed
Feseha et al., 2012^44^	Community-based cross-sectional study	SRH including GBV; Maternal and neonatal health Physical violence for two timeframes: 12 months preceding interview; any time during the woman’s life since she started relationship with the current partner. Data from pregnant women also included	Prevalence of physical violence	Northern Ethiopia	1223 women of reproductive age	Peer reviewed
Ghazi et al., 2013^45^	Cross-sectional self-administered questionnaire	Infant and child health Anthropometric measurements and family social factors	Identified factors associated with child malnutrition	Iraq – conflict	220 children aged between 3–5 years	Peer reviewed
Gitau et al., 2005^46^	Longitudinal cohort study, standardized questionnaire, physical examination and laboratory tests	Maternal and neonatal health; Infant and child health Vitamin A during pregnancy, Vitamin E post-partum, maternal weight and haemoglobin; infant length and weight	Effects of drought on maternal and infant health	Zambia – drought and famine	429 women of reproductive age	Peer reviewed
Gordon & Halileh, 2013^47^	Cross-sectional survey using WHO child growth standards	Infant and child health Anthropometric measurements; birth weight; breastfeeding practice, family and household social factors	Identified factors associated with child stunting	West Bank and Gaza strip – conflict	9051 children younger than five years	Peer reviewed
Guerrier et al., 2009^48^	Two stage cluster survey	Infant and child health Anthropometric indices and measles vaccination history	Crude mortality rate, under-five mortality rate, prevalence of wasting and vaccination status among children aged between 6 months and 5 years	Eastern Chad – IDP	80 300 individuals	Peer reviewed
Hapsari et al., 2009^49^	Community based surveys	SRH including GBV Access to contraception, change in contraceptive methods before and after the earthquake, prevalence of unplanned pregnancy	To plan for effective family planning coverage	Indonesia – earthquake	450 women of reproductive age	Peer reviewed
Helweg-Larsen et al., 2004^50^	Data collection from medical records using ICD-10 and International Classification of External Causes of Injuries	Infant and child health Intent, mechanism, means, context and place of intentional injuries among children, relationship with perpetrator	To evaluate the combination of ICD– 10 and International Classification of External Causes of Injuries, to test the feasibility of a systematic documentation of public health consequences of such conflicts	West Bank and Gaza strip – uprising	NA	Peer reviewed
Hossain et al., 2009^51^	Cross-sectional household survey using clusters; No information provided for tool	Infant and child health Prevalence of acute malnutrition in children	To identify the relationship between food aid and nutritional status	Pakistan – earthquake	1114 children aged between 6 and 59 months	Peer reviewed
Hudson et al., 2010^52^	Semi-structured questionnaire containing quantitative and open-ended questions	SRH including GBV; Maternal and neonatal health; STI including HIV; Access to medical care, access to care during pregnancy and childbirth, access to food, water, and hygiene facilities, perception of personal safety	Needs assessment	Haiti – post earthquake with long-term political instability, IDP camp	64 women of reproductive age	Not peer reviewed
IRC et al., 2003^53^	Interview questionnaire	SRH including GBV Demographic characteristics of women	To estimate the prevalence of GBV in women and the consequences of such violence on mental, sexual and RH	Colombia – IDP from internal conflict	NA	Not peer reviewed
Jayatissa et al., 2006^7^	Cross-sectional, two-stage cluster, rapid assessment nutrition survey, interviewer administered questionnaire, anthropometrics, FGDs and KIIs	Maternal and neonatal health; Infant and child health Prevalence of acute and chronic malnutrition in children and under-nutrition among pregnant and lactating women	For policy recommendation regarding setting up of nutritional surveillance systems	Sri Lanka – 42 tsunami relief camps	875 children younger than five years; 168 pregnant women, 97 lactating women	Peer reviewed
JSI Research & Training Institute, 2002^54^	Questions from reproductive health response in crises and refugee reproductive health needs assessment field tools used in group discussions	SRH including GBV; Maternal and neonatal health; STI including HIV Status and availability of services regarding safe motherhood, family planning, SGBV, adolescent sexual and reproductive health, STIs/HIV	To assess the RH needs and RH services	Democratic Republic of the Congo – IDP population in civil war	NA	Not peer reviewed
JSI Research & Training Institute, 2009^55^	Interviews and in-depth discussions with snowball sampling; no specific tools described	SRH including GBV; Maternal and neonatal health; STI including HIV Accessibility and availability of services regarding safe motherhood, family planning, SGBV, STIs/HIV	To identify gaps in the availability and accessibility of comprehensive RH services	Haiti – hurricanes	NA	Not peer reviewed
Kalter et al., 2008^56^	Standardized questionnaire based on verbal autopsy formats; prospective monitoring of pregnant women and newborns from randomly selected clusters	Maternal and neonatal health Causes of neonatal and perinatal deaths, neonatal and perinatal mortality rates, including still births	To identify risk factors for perinatal deaths	West Bank and Gaza strip – uprising	926 women of reproductive age	Peer reviewed
Khalidi et al., 2004^57^	Stratified random sampling of 301 households (2025 families); Person-to-person interviews, household questionnaires and individual questionnaires	SRH including GBV Knowledge, attitudes and practice of domestic violence recognition, management and prevention	Recommendations for the next steps of the project aimed at better understanding factors related to the severity of the domestic violence problem	Lebanon – refugee camps	2018 individuals	Not peer reviewed
Kottegoda et al., 2008^58^	Interviews and structured questionnaire	SRH including GBV; Maternal and neonatal health RH concerns (early marriage, early pregnancy, miscarriage, home births and GBV)	To highlight the voices of women who were shadowed by conflict	Sri Lanka – conflict	560 women aged 12–60 years	Peer reviewed
Krause et al., 2003^59^	Reproductive health response in crises Reproductive Health assessment toolkit	SRH including GBV; Maternal and neonatal health; STI including HIV MISP services availability (sexual and gender based violence, family planning, safe motherhood, STI/HIVs)	Data used for formulating policy recommendations	Colombia	363 individuals	Not peer reviewed
Krause et al., 2011^60^	MISP assessment using reproductive health response in crises toolkit	SRH including GBV; Maternal and neonatal health; STI including HIV awareness about the need for MISP among international organizations; effectiveness of early disaster response; coordination of anti-GBV effort; availability of HIV/AIDS management, family planning, ANC and emergency obstetric care	Assessment on effectiveness of SRH service delivery	Haiti – post-earthquake with long-term political instability		Not peer reviewed
Lederman et al., 2008^61^	Interview; material hardship scale	Maternal and neonatal health data on maternal medical, obstetrics; birth weight, heights, head circumference and gestational duration	Relationship of perceived air pollution and modelled air pollution to maternal characteristics and birth outcomes	USA – 400 different locations	NA	Peer reviewed
Lee, 2008^62^	KII with health care professionals from NGO and government facilities	SRH including GBV; Maternal and neonatal health Type of reproductive health service provision, delivery pattern, security issues of the service providers	To explore the availability of services provided in long-standing internal conflict	Maguindanao, Philippines	8 individuals	Peer reviewed
Longombe et al., 2008^63^	Review of hospital records of victims of sexual violence	SRH including GBV; including HIV Prevalence of fistula, sexually transmitted diseases	Basis for formulating policy recommendations to develop a coordinated efforts among key stakeholders	Democratic Republic of the Congo – armed conflict and post conflict	7 survivors	Peer reviewed
Mason et al., 2005^64^	Child anthropometry and survey with two-stage cluster sampling	Maternal and neonatal health; Infant and child health Prevalence of underweight	Results of child malnutrition in six countries in southern Africa	Lesotho, Malawi, Mozambique, Swaziland, Zambia and Zimbabwe – severe drought	NA	Peer reviewed
Mateen et al., 2012^65^	Data collected from the United Nations refugee assistance information system, ICD-10	Infant and child health Common neurological disorders	Diagnosis of common neurological disorders in refugees (men and women)	Jordan –refugees from Iraq	31 476 individuals	Peer reviewed
Mateen et al., 2012^66^	Data collected from the United Nations refugee assistance information system	Maternal and neonatal health; Infant and child health Communicable and noncommunicable diseases, health service utilizations	Determining the range infections and burden of health services use among adults and children (0–17years)	Jordan – refugees from Iraq	7642 individuals	Peer reviewed
McGinn et al., 2001^67^	Interviews and self-administered questionnaires	SRH including GBV Acceptance of contraceptive methods by women; FP policies and management systems from organizations	Six specific recommendations were formulated	Pakistan – Afghan refugee camps	NA	Not peer reviewed
Minetti et al., 2009^68^	Medecins Sans Frontieres programme monitoring data (medical records), physical examination	Infant and child health Weight, height and length of children, presence of oedema	Evaluation of the change from National Center for Health Statistics to WHO 2006 growth standards children (6m-5y). Led to identification of a larger number of malnourished children at an earlier stage	Niger – severe malnutrition	NA	Peer reviewed
Mullany et al., 2008^69^	Population-based, cluster-sample surveys, FGDs, pregnancy records	SRH including GBV; Maternal and neonatal health; Infant and child health Basic demographics, obstetric history, human right violations	Monitoring and evaluation of MOM project in delivering maternal health services by qualitative and quantitative methods	Myanmar – IDP and conflict	59,042 individuals	Peer reviewed
Murray et al., 2009^70^	Study specific rapid health assessment tool (included), interviews	Infant and child health Surveillance of infectious diseases in hurricane evacuees	To identify potential disease outbreaks	USA – hurricane	29 478 individuals	Peer reviewed
Nichols et al., 2013^71^	Rapid assessment, mass screening, and convenience sample	Infant and child health Biochemical analysis of riboflavin from children and adults	To provide guidelines for monitoring micronutrient deficiency in adults and children receiving food assistance	Uganda – drought	179 172 individuals	Peer reviewed
Noe et al., 2013^72^	Retrospective aggregate of routine data collection, including the disaster health services aggregate morbidity report form	Maternal and neonatal health, Infant and child health Data on immediate medical needs of evacuees following hurricanes	To identify health care delivery needs during a relief operation	USA – hurricane	3863 individuals	Peer reviewed
Nsuami et al., 2013^73^	Cross-sectional, survey	STI including HIV Urine screening for gonorrhoea and chlamydia in high schools	Prevalence of gonorrhoea and chlamydia before and after hurricane with the suggestion for STI screening immediately after natural disasters	USA – hurricane	679 individuals	Peer reviewed
Patel et al., 2014^74^	Cross-sectional demographic and behavioural survey	STI including HIV testing; sexual behaviour	Identified risk factors for HIV infection	Uganda – post-conflict transit camp	384 adolescents	Peer reviewed
Physicians for Human Rights, 2009^75^	Quantitative and qualitative data from a non-probability sample, questionnaire, physical and psychological evaluation, interviews with stakeholders	SRH including GBV Physical and psychological consequences of rape and exposure to extreme violence	Provide insight into the experiences and suffering and provided a basis for recommendations	Border between Chad and Sudan – refugee camps	88 women	Not peer reviewed
Ravindranath et al., 2005^76^	Household survey using cluster sampling, anthropometry and physical examination	Infant and child health Underweight in school children, chronic energy deficiency in adults assessed by body mass index	Assessment of nutritional status of community during drought and also evaluation of coping mechanisms by the intake of food and nutrient intakes	India – severe drought	NA	Peer reviewed
RHRC, 2004 AMDD^77^	Facility assessment; AMDD tool	Maternal and neonatal health Availability of emergency obstetric care services	To establish and improve basic and comprehensive emergency obstetric care services at health centres and hospitals responding to emergency obstetric needs of refugees and others of reproductive age living within and around the refugee community	Bosnia and Herzegovina, Kenya, Liberia, Pakistan, Sierra Leone, Sudan, Tanzania, Thailand and Uganda	NA	Not peer reviewed
RHRC, 2006 AMDD Program^78^	Facility assessment; AMDD tool	Maternal and neonatal health Availability of emergency obstetric care services	Monitoring and evaluation of basic emergency obstetric care at the health centre level and comprehensive emergency obstetric care at the hospital level was carried out to review emergency obstetric service delivery protocols	Bosnia and Herzegovina, Kenya, Liberia, Pakistan, Sierra Leone, Sudan, Tanzania, Thailand and Uganda	NA	Not peer reviewed
Rodriguez et al., 2006^79^	Survey using study specific questionnaire modelled after previous post-disaster surveys (EpiInfo3.2.2)	Infant and child health Individual on pre-existing medical and household characteristics	To determine medical and social needs to allocate resources	USA –post–hurricane	371 individuals	Peer reviewed
Saile et al., 2013^80^	Survey; structured interviews, standardized questionnaires, composite abuse scale, violence, war and abduction exposure scale, posttraumatic diagnostic scale; depression – Hopkins symptom checklist, alcohol use disorder identification test	SRH including GBV Frequency and types of abuse experienced	Described partner abuse and predictor variables	Uganda – post-conflict	470 individuals	Peer reviewed
Salama et al., 2001^81^	Two-stage cluster survey, standardized questionnaire	Infant and child health Crude mortality and mortality of children younger than five years, causes of death and anthropometric measurements	To estimate major causes of deaths and prevalence of malnutrition among children and adults	Ethiopia – famine	4032 individuals	Peer reviewed
Sawalha et al., 2013^82^	Cross-sectional survey; sociodemographic questionnaire, laboratory test	Infant and child health Blood lead levels; sociodemographics; general health	Assessed blood lead levels	West Bank and Gaza strip – refugee camp	178 children aged 6–8 years	Peer reviewed
Sherrieb & Norris, 2012^83^	Review of birth outcomes pre- and post-event	Maternal and neonatal health Birth weight and preterm births	Impact of terrorist attacks on population health	USA – terrorist attack	NA	Peer reviewed
Spiegel et al., 2014^84^	Surveillance survey; descriptive data analysis, multivariable logistic regression	Maternal and neonatal health Sexual history and behaviour, HIV knowledge and testing, refugee type and length, interaction between groups	Identified factors independently associated with multiple sexual partnerships	Botswana, Kenya, Mozambique, Nepal, Rwanda, South Sudan, Sudan, Tanzania, Uganda – refugees	24 219 individuals	Peer reviewed
Sullivan et al., 2004^85^	Adapted reproductive health response in crises Reproductive health needs assessment field tools	Maternal and neonatal health Data on catchment area, SRH service availability and coverage including staffing, equipment and supplies, client perception	To improve RH and building clinic capacity in monitoring and evaluation	Border between Myanmar and Thailand – illegal immigrant workers and IDPs	462 women	Peer reviewed
Talley & Boyd 2013^86^	Retrospective record review; standardized, study specific, data collection tool	Maternal and neonatal health Demographics, admission criteria, primary caretaker, infant feeding practices, anthropometrics	Evaluation of infant feeding programme	Haiti – earthquake	493 infants	Peer reviewed
Tan et al., 2009^87^	Analysis of birth records	Maternal and neonatal health Birth weight, APGAR score, pre- and post-event	Effects of earthquake on birth outcomes	China – earthquake	13 003 neonates	Peer reviewed
Tappis H et al., 2012^88^	Secondary data analysis of UNHCR Twine database	Infant and child health Growth and nutrition data on the refugee camp population	Effectiveness of the coverage of UNHCR supplementary and therapeutic feeding programmes for the malnourished children	Kenya and Tanzania –refugees	39 899 children younger than five years	Peer reviewed
Teela et al., 2009^89^	FGDs and detailed case studies with maternal health workers; no specific tools described	11=SRH including GBV,2; 2=Maternal and neonatal health Characteristics of maternal health workers in conflict settings, their efforts on community mobilization, provision of emergency obstetric care and technical competence, security and logistical constraints, programme successes	To complement project quantitative information and provide contextual information of the community maternal health workers’ challenges in implementation	Eastern Myanmar – conflict	41 health workers	Peer reviewed
Tomczyk et al., 2007^90^	Population-based survey of a sample of 36 primary sampling units; CDC RH assessment toolkit	SRH including GBV; Maternal and neonatal health; STI including HIV Social background, maternal health, contraception, violence; HIV/AIDS knowledge, attitudes, and risk behaviours	Policy recommendations regarding continuous funding when traditional humanitarian aid is limited or withdrawn	Liberia – post-protracted armed conflict and transitional years	907 women of reproductive age	Not peer reviewed
Turner et al., 2013^91^	Informal staff interviews	Infant and child health Admission diagnosis and characteristics, treatment provided	Impact of introduction of special care baby unit on refugee population	Myanmar – refugees	952 infants	Peer reviewed
Turner et al., 2013^92^	Laboratory-enhanced, hospital-based surveillance; Patient interview, record review	Infant and child health Patient symptoms, nasopharyngeal aspirates, pyrexia, respiration rate	Characterization of the epidemiology of respiratory virus infections in refugees	Border between Myanmar and Thailand – refugees	635 children younger than five years and 68 children older than 5 years	Peer reviewed
UNHCR et al., 2011^93^	Health facility assessment, IDIs, FGDs and household surveys; CDC RH assessment tool	SRH including GBV Knowledge, beliefs, perceptions and practices surrounding family planning	To improve programming and subsequently increase uptake of good quality family planning services	Kenya – refugees from Somalia	NA	Not peer reviewed
UNHCR et al., 2011^94^	Health facility assessment, IDIs, FGDs and household surveys; CDC RH assessment tool	SRH including GBV Knowledge, beliefs, perceptions and practices surrounding family planning	To improve programming and subsequently increase uptake of good quality family planning services	Jordan – refugees from Iraq	NA	Not peer reviewed
UNHCR et al., 2011^95^	Health facility assessment, IDIs, FGDs and household surveys; CDC RH assessment tool	SRH including GBV Knowledge, beliefs, perceptions and practices surrounding family planning , the state of service provision	To improve programming and subsequently increase uptake of good quality family planning services	Djibouti – refugees from Somalia	NA	Not peer reviewed
UNHCR et al., 2011^96^	Health facility assessment, in-depth interviews, focus group discussions and household survey; CDC RH assessment tool	SRH including GBV; Knowledge, beliefs, perceptions and practices surrounding family planning, the state of service provision	To improve programming and subsequently increase uptake of good quality family planning services	Uganda – refugees from the Democratic Republic of Congo	NA	Not peer reviewed
UNHCR et al., 2011^97^	Health facility assessment, in-depth interviews, focus group discussions and household survey; CDC RH assessment tool	SRH including GBV Knowledge, beliefs, perceptions and practices surrounding family planning, the state of service provision	To improve programming and subsequently increase uptake of good quality family planning services	Malaysia – refugees from Myanmar	NA	Not peer reviewed
Usta et al., 2010^98^	The international child abuse screening tool (International Society for the Prevention of Child Abuse and Neglect (IPSCAN-2007) was translated from English into Arabic	SRH including GBV Child sexual abuse pre and post-conflict	The prevalence, risk factors and consequences of child sexual abuse in Lebanese children	Lebanon	1028 children aged between 8–17 years	Peer reviewed
Wainstock et al., 2013^99^	Retrospective cohort study; Interviews	Maternal and neonatal health sociodemographics, smoking, perceived stress, clinical data from hospital records	Evaluation of the association between prenatal maternal stress and preterm birth and low-birth weight	Israel – conflict (rocket attacks)	125 women	Peer reviewed
Ward, 2002^100^	Interviews with IDP and actors; no specific tools described	SRH including GBV Overview of GBV findings globally	To inform of services available and programming gaps relating to gender based violence in conflict-affected populations	Border between Afghanistan and Pakistan, Azerbaijan, Bosnia and Herzegovina Democratic Republic of the Congo, border between Myanmar and Thailand Rwanda, Sierra Leone, Timor Leste, – conflict affected populations	NA	Not peer reviewed
Wayte et al., 2008^101^	IDI, service statistics and document review; No specific tool described	SRH including GBV; Maternal and neonatal health; STI including HIV RH service provision, coordination and priority setting; ANC; Maternity waiting home; Family planning; STIs, HIV/AIDS; Gender based violence, adolescent health	To assess the health sector’s response to RH	Timor Leste	35 individuals	Peer reviewed
Wilson et al., 2013^102^	Retrospective review of paediatric registry records	Infant and child health Demographics, mechanism of injury, clinical and laboratory data, diagnostic and surgical procedures, complications and outcomes	Review of paediatric trauma in a combat support hospital	Afghanistan – conflict	41 children aged between 1–18 years	Peer reviewed
Wirtz et al., 2013^103^	IDIs, FGDs	SRH including GBV; Prevalence of GBV, physical and psychological consequences of GBV	To inform the development of a screening tool as a potential strategy for addressing GBV	Ethiopia – refugees from Somalia, post-conflict	144 individuals	Peer reviewed
Women’s Commission, 2002^104^	Reproductive health needs assessment field tools	SRH including GBV; Maternal and neonatal health; STI including HIV Status and availability of services regarding safe motherhood, family planning, SGBV, adolescent SRH, STIs/HIV	To assess RH	Zambia – civil war refugees from Angola and Democratic Republic of Congo	NA	Not peer reviewed
Women’s Commission, 2003^105^	Based upon RHRC toolkit	SRH including GBV; Maternal and neonatal health; STI including HIV Family planning, SGBV, Adolescent SRH, safe motherhood, STI, HIV; Availability of instructional resource materials	Data for policy recommendations and to identify their problems in assessing the services	Pakistan – Refugees from Afghanistan	NA	Not peer reviewed
Women’s Commission, UNFPA, 2004^106^	Semi-structured interview, FGD, and health facility assessment; MISP assessment tool kit	SRH including GBV; Maternal and neonatal health; STI including HIV Status and availability of services under MISP; Coordination among RH service providers	To evaluate the implementation of the MISP and the use of RH kits	Chad – refugees from South Sudan	108 individuals	Not peer reviewed
Women’s Commission, 2005^107^	Cross sectional, interviews and FGD, No specific tools described	SRH including GBV, Maternal and neonatal health STI including HIV Status and availability of services under MISP; Coordination among RH service providers	To assess the implementation of MISP activities, and the agency staffs’ understanding of MISP	Indonesia – tsunami	77 individuals	Not peer reviewed
Women’s Commission, 2007^108^	Structured interviews, meetings with representatives of local and international NGOs, 10 focus groups with displaced persons; visits to local facilities	SRH including GBV; Maternal and neonatal health; STI including HIV SRH service availability and use in family planning, SGBV, safe motherhood, STIs and HIV/AIDS	Basis for formulating recommendations regarding: funding, coordination, staffing, training, RH equipment and supplies, safe motherhood, FM, STIs and GBV	Northern Uganda – protracted civil war	140 females and youths	Not peer reviewed
Women’s Commission, 2008^109^	Cross sectional, interviews, FGD and observations. MISP	SRH including GBV; Maternal and neonatal health; STI including HIV Sexual violence, HIV, maternal and newborn morbidity and mortality	The purpose of the assessment was to examine the degree of implementation of the MISP for RH	Kenya	139 individuals	Not peer reviewed
Women’s Wellness Centre & RHRC, 2006^110^	Household survey of women of reproductive age	SRH including GBV; Estimates of sexual and physical violence prevalence	Data obtained used for formulating policy recommendations	Nine villages in Peja region, Serbia – conflict, displacement and post-conflict setting	332 women of reproductive age	Not peer reviewed

AMDD: Averting Maternal Death and Disability, ANC: Antenatal Care, ARI: Acute Respiratory Infection, BMI: Body Mass Index, CDC: Centers for Disease Control, FGD: Focus Group Discussions, FP: Family Planning, GBV: Gender Based Violence, HIV**:** Human Immunodeficiency Virus, ICD-10: International Classification of Diseases 10^th^ edition, IDI: In-depth Interview, IDP: Internally Displace People, IPV: Intimate Partner Violence, KII: Key Informant Interviews, M&E: Monitoring and Evaluation, MISP: Minimum Initial Service Package, NA: not available, NGO: Nongovernmental organizations, PNC: Postnatal care, RH: Reproductive Health, RHRC: Reproductive Health Response in Crises Consortium, RTI: Reproductive Tract Infections, SGBV: Sexual and Gender Based Violence, SRH: Sexual and Reproductive Health, STI: Sexually Transmitted Infection, U5: Under five years of age

Data were collected from refugee populations in the recovery phase. Our review did not identify any studies that collected data during the disaster preparedness phase, which is defined by UNFPA as, “the period preceding a humanitarian crisis – use of early warning signals to avert crises or prepare response”.^111^ Seventy-six studies examined the health status of the population affected, while 24 examined the availability and coverage of health services, usually measured using the minimum initial service package.^60^ A variety of indicators were collected with some studies using specific toolkits for field settings (Table 2).

**Table 2:** Data collection tools used and type of data collected for maternal and child health during humanitarian emergencies

Category	Type of data collected	Tool application described in the literature
**Sexual and reproductive health**		
Family planning^28^^,^^35^^,^^49^^,^^52^^,^^54^^,^^55^^,^^58^^–^^60^^,^^62^^,^^67^^,^^69^^,^^93^^–^^97^^,^^101^^,^^104^^–^^109^	SRH including MNCH, availability and accessibility of modern contraceptives, couple discussion on methods of choice, unplanned pregnancy, knowledge, attitude and practices of family planning, security of family planning.	CDC RH assessment toolkit for conflict-affected women, RHRC RH needs assessment field tools, MISP assessment
Sexual and gender-based violence ^15^^,^^16^^,^^22^^,^^24^^,^^25^^,^^33^^,^^37^^,^^39^^,^^43^^,^^44^^,^^53^^–^^55^^,^^58^^–^^60^^,^^63^^,^^75^^,^^80^^,^^90^^,^^98^^,^^100^^,^^101^^,^^103^^–^^110^^,^^112^	Prevalence of child sexual abuse, risk factors of sexual and gender-based violence, patterns of sexual and gender-based violence, awareness among aid workers of sexual and gender-based violence, efficiency of response and coordination among agencies, availability and accessibility of services for sexual and gender-based violence victims, intimate partner violence and associated factors, physical consequences of sexual and gender-based violence (fistula and infections), mental consequences.	MISP assessment toolkit, AUDIT (The Alcohol Use Disorders Identification Test: Guidelines for Use in Primary Care), Measuring Intimate Partner Violence Victimization and Perpetration: A Compendium of Assessment Tools (CDC, 2006).
**Maternal and newborn health**		
Emergency Obstetric Care^34^^,^^60^^,^^77^^–^^79^	Number of deliveries at health facilities, caesarean section rate, availability of blood transfusion, obstetric complications managed, manual vacuum aspiration procedures performed, maternal deaths.	Emergency obstetric and newborn care assessment toolkit from the Averting Maternal Death and Disability (AMDD) programme.
Newborn health^46^^,^^56^^,^^83^^,^^87^^,^^91^	Birth outcomes, birth defects.	No description of specific tools used.
General maternal and newborn health ^7^^,^^12^^,^^18^^,^^21^^,^^27^^,^^28^^,^^37^^,^^38^^,^^43^^,^^44^^,^^46^^,^^52^^–^^55^^,^^58^^–^^62^^,^^64^^,^^66^^,^^69^^,^^72^^,^^85^^–^^87^^,^^90^^,^^99^^,^^101^^,^^104^^–^^110^	Logistics and security issues, antenatal care, maternal height and weight, vitamin A during pregnancy, iron and folate supplementation, malaria during pregnancy, anaemia during pregnancy, human rights violations, barriers to receiving care.	RHRC RH needs assessment field tools, MISP assessment toolkit.
**Infant and child health**		
Nutrition^7^^,^^12^^,^^13^^,^^20^^,^^21^^,^^30^^,^^36^^,^^45^^–^^48^^,^^51^^,^^64^^,^^68^^,^^69^^,^^71^^,^^72^^,^^76^^,^^79^^,^^81^^,^^82^^,^^88^	Weight, height and mid upper arm circumference (MUAC) of children, vaccination status of children, presence of oedema, haemoglobin levels, other infections (acute respiratory infections, diarrhoea), other nutritional and micronutrient deficiency, feeding practices (exclusive breastfeeding, complementary feeding), food assistance and food security.	No description of specific tools used.
Infections^12^^,^^14^^,^^19^^,^^26^^,^^30^^,^^32^^,^^42^^,^^66^^,^^70^^,^^92^	Socioeconomic factors, demographic factors, diarrhoea and waterborne infections, acute respiratory infections and diseases of adenoids, visual disturbances, urinary problems, malaria treatment and use of insecticide-treated nets.	No description of specific tools used.
Injuries^29^^,^^38^^,^^40^^,^^41^^,^^50^^,^^102^	Types of injuries, care seeking behaviour, intentional injuries including context, when and how it occurred, weapon used, relationship with perpetrator, injuries by landmines and unexploded ordinances (time, place and how it happened, type and site of injury), need for blood transfusion	No description of specific tools used.
Miscellaneous^31^^,^^46^^,^^47^^,^^65^	Lead poisoning (blood-lead level, chelation therapy), medical health conditions, mental child health conditions, neurological disorders including epilepsy, infantile cerebral palsy.	No description of specific tools used.
**Sexually transmitted infections including human immunodeficiency virus infection (HIV) and acquired immunodeficiency syndrome (AIDS)**^17^^,^^23^^,^^28^^,^^33^^,^^39^^,^^52^^,^^54^^,^^55^^,^^59^^,^^60^^,^^63^^,^^73^^,^^74^^,^^90^^,^^101^^,^^104^^–^^109^	Availability and accessibility of HIV/AIDS management, knowledge and attitudes on HIV/AIDS, risk behaviour on HIV/AIDS, prevalence of sexually transmitted infections as consequence of sexual and gender based violence, availability of resource materials for sexually transmitted infections and HIV, prevalence of gonorrhoea and chlamydia.	MISP assessment toolkit.

AIDS: acquired immunodeficiency syndrome; CDC RH: Centers for Disease Control and Prevention, Reproductive Health; HIV: human immunodeficiency virus; MISP: minimum initial service package; MNCH: maternal, newborn and child health; RH: reproductive health; RHRC: reproductive health response in conflict; SRH: sexual and reproductive health

Data were collected for monitoring and evaluation purposes in 69 studies. In 18 studies, data were collected for the purpose of advocacy; seven studies were operational research and six studies described a needs assessment. No studies that we identified had the primary aim of collecting data to support a funding request.

### Data collection tools

We identified a total of 17 different tools which were mainly structured questionnaires (Table 3). Among 100 included studies, 19 specified the use of any of the 17 identified tools. Eight studies used a rapid assessment field tool;^55^^,^^59^^,^^60^^,^^85^^,^^104^^–^^106^^,^^109^ seven used the assessment toolkit for conflict affected women^35^^,^^90^^,^^93^^,^^94^^–^^97^ and three used the emergency obstetric care assessment toolkit from the averting maternal disability and deaths programme.^34^^,^^77^^,^^78^ The alcohol use disorders identification test;^112^ the compendium for measuring intimate partner violence victimization and perpetration^122^ and Twine (a web-based toolkit developed by the Office of the United Nations High Commissioner for Refugees)^4^ were used in one study each. The remaining 79 studies did not specify which tools had been used to collect the data.

**Table 3:** Summary of data collection tools for maternal and child health in humanitarian emergencies, by year of publication

Existing tools for data collection identified from the literature review	Type of data that can be collected				Suitable in acute phase of an emergency	Field application reported
	Sexual & reproductive health including gender-based violence	Maternal and newborn health	Infant and child health	Sexually transmitted infections		
Twine (United Nations High Commissioner for Refugees, 2014)^4^	Yes	Yes	Yes	Yes		Yes
Refugee health: an approach to emergency situations^a^ (Médecins Sans Frontières, 1997)^113^		Yes	Yes	Yes		
Refugee RH needs assessment field tools (Reproductive Health Response in Crises Consortium, 1997)^114^^–^^117^	Yes	Yes		Yes		Yes
The alcohol use disorders identification test: guidelines for use in primary health care (Babor, 2001)^112^	Yes					Yes
SGBV Tools for refugees, returnees and IDPs (United Nations High Commissioner for Refugees, 2003)^118^	Yes			Yes		
EmOC needs assessment tool (Women’s Commission and Averting Maternal Death and Disability, 2005)^119^		Yes				Yes
GBV prevention and response tool in emergencies (Inter-Agency Standing Committee, 2005)^120^	Yes				Yes	
Guidelines on public health promotion in emergencies (Oxfam, 2006)^121^	Yes				Yes	
Measuring intimate partner violence victimization and perpetration: a compendium of assessment tools (Centers for Disease Control and Prevention, 2006)^122^	Yes					Yes
Adolescent SRH toolkit for humanitarian settings (United Nations Population Fund and Save the Children Fund, 2010)^123^	Yes			Yes		
GBV programme monitoring tool, (United Nations Population Fund, 2010)^124^	Yes					
Inter-agency field manual on RH in humanitarian settings (WHO Interagency Working Group on Reproductive Health in Crises, 2010)^125^	Yes					
MISP assessment toolkit (Interagency Working Group on Reproductive Health in Crises, 2010)^126^	Yes	Yes		Yes	Yes	
RH assessment toolkit for conflict-affected women, (Centers for Disease Control and Prevention, 2011)^127^		Yes				Yes
Sphere handbook (The Sphere Project, 2011)^128^	Yes	Yes	Yes	Yes		
Guide to MNCH and nutrition in emergencies (World Vision, 2012)^1^	Yes	Yes	Yes		Yes	
GBV tools manual for assessment and program design, monitoring and evaluation in conflict-affected settings (Reproductive Health Response in Crises Consortium, 2005)^129^	Yes					

EmOC: emergency obstetric care; GBV: gender-based violence; IDP: internally displaced persons; MISP: minimum initial service package; MNCH: maternal, newborn and child health; RH: reproductive health; RHRC: reproductive health response in crises consortium; SGBV: sexual and gender-based violence; SRH: sexual and reproductive health; WHO: World Health Organization.

^a^ General toolkits that do not exclusively assess SRH or MNCH.

Of the 17 toolkits identified (Table 3), 14 could be used to collect data on sexual and reproductive health, eight on maternal and newborn health, four on child health and seven on sexually transmitted infections and HIV. Some of the tools were designed to collect more than one category of data (e.g. Twine). Of the 14 tools used for data collection on sexual and reproductive health, four were specifically designed for gender-based violence. A further 13 studies also collected data on gender-based violence, but no data collection tool was identified.

Similarly, there was no specific tool to collect child health data, but four toolkits had questionnaires that included the collection of some data on child health data. Twine contains a specific section for child health data collection, including nutrition.^4^ *Refugee health: an approach to emergency situations*^113^ is designed to collect data on children for diseases under surveillance, nutritional status and common communicable diseases. The Sphere handbook^128^ has rapid assessment tools to collect health service assessment data as well as sample surveillance reporting forms. These can be used to collect information on children younger than five years and provide outbreak alerts for this age group. These tools incorporate early warning and response network surveillance for early detection of epidemic-prone diseases in emergency settings. We did not identify specific tools for sexually transmitted infections and HIV, but relevant data are collected as part of seven of the more general sexual and reproductive health toolkits.^130^

## Discussion

Our review provides an overview of the data collection tools available as well as the published experience of the use of these tools. We advocate the use and harmonization of existing tools rather than the development of new tools. As we could not identify any studies reporting on data collection for disaster preparedness or disaster response, there is a need to adapt existing tools or develop new tools to facilitate data collection specifically for these phases. We excluded tools used primarily in non-humanitarian settings and may not have captured all available tools or data collected in humanitarian emergency settings.

Most of the tools specify which methods are needed to collect the required data, including both quantitative and qualitative methods in specific contexts. The methods used depend upon the purpose of data collection, the available resources and the nature of the information sought. Table 4 summarizes commonly reported methods to collect data during an emergency.^130^

**Table 4:** Approaches and methods for the collection of data during humanitarian emergencies

Approach	Methods	Data sources
Qualitative	Key informant interviews	Key stakeholders (e.g. health service providers, policy- and decision-makers)
	Focus group discussions	Affected population
Mixed Method	Observational study	Affected population and area
	Inventory or document review	Previous available data (e.g. surveys, health sector data, programme reports)
Quantitative	Secondary data analysis	Previous available data (e.g. surveys, health sector data, programme reports)
	Rapid counting	Affected population
	Aerial surveillance	Affected area
	Flow monitoring	Affected population
	Enumeration or profiling	Affected population

Of the 100 studies included in this review, only 19 described the data collection tools used and only six commented on their applicability in field settings. Authors may not be aware of the existence of a wide range of toolkits, or the importance of documenting their experiences.

To improve the response to humanitarian emergencies, target groups need to be identified and their specific needs understood. For sexual, reproductive, maternal, newborn and child health the underlying contexts which prevent or enable access to services also need to be considered.^130^ The international humanitarian community continues to highlight the importance of documenting and addressing the problem of sexual and gender-based violence.^37^ A central repository of data collected during a humanitarian emergency, where a core set of indicators is agreed on, would be useful. The repository would allow any user to submit or explore data to inform decision-making and enable comparisons between and across settings.

Only eight studies were conducted within the first six months of a humanitarian emergency. The majority of studies (69/100) and data collected were used to monitor and evaluate ongoing interventions. This may reflect the necessity of providing immediate life saving measures during the early stages of humanitarian emergencies. Rapid assessments are vital in the early stages of humanitarian emergencies. Information is required to highlight changing needs to inform appropriate provision of relief and urgent medical assistance. Most importantly, rapid assessment tools need to be simple to use.^131^

It is encouraging to note that the tools developed so far seem to have used a cluster approach for data collection. Introduced in 2006 as part of the UN Humanitarian Response, a cluster is defined as:

“a group of agencies that gather to work together towards common objectives within a particular set of emergency response”.^132^

The approach aims to improve the effectiveness of humanitarian assistance by improving predictability and timeliness of a response process through a coordinated effort.^111^ The cluster approach can strengthen accountability among key actors and enhance the complementary nature of different organizations involved in providing humanitarian assistance. Although the health and nutrition clusters are critical for maternal, newborn and child health, the available tools consider other clusters as cross-cutting areas including protection, water and sanitation, camp coordination and management.^132^

## Conclusion

There is a need to evaluate, standardize and harmonize existing data collection toolkits and to develop others that can be used in the response phase of humanitarian emergencies. Information is needed on the applicability of existing tools in relation to the types of populations and the emergency situations in which they are used. It would be useful to develop shortened versions of existing tools adapted specifically to use in the response phase, together with a more comprehensive version for the later phases of an emergency. Humanitarian assistance reports should include analyses of the lessons learnt when using data collection toolkits. This information can assist modification of existing tools and development of new tools. Whenever new toolkits are developed by interagency working groups, it is important to take the perspectives of field users into account. Wider dissemination of the availability of data collection tools among humanitarian workers can be achieved by educating staff at headquarters and country offices of humanitarian organizations, or by including the toolkits in disaster risk reduction training.

To plan and evaluate interventions and actions that will save lives in humanitarian emergencies, appropriate data are needed. To ensure that tools used to obtain such data are easy to use and comprehensive, it is essential that both individuals involved in field operations and in operations research continue to work together. New standardized tools should be developed and existing ones adapted based upon standards for data collection in emergencies with inputs from humanitarian agencies.^111^ This work could be coordinated by WHO.
